# Optimizing Optical Film Lamination to Enhance the Luminance of TFT-LCD Displays Using the Taguchi-NNGA Method

**DOI:** 10.3390/ma14164481

**Published:** 2021-08-10

**Authors:** Yungho Leu, Chia-Ming Lin

**Affiliations:** Department of Information Management, National Taiwan University of Science and Technology, 43, Keelung Road, Section 4, Taipei 10607, Taiwan; yhl@cs.ntust.edu.tw

**Keywords:** laminated materials, TFT-LCD displays, Taguchi method, neural network, genetic algorithm

## Abstract

Luminance is an essential quality of a TFT-LCD display. Manufacturers have attempted to improve the soft-to-hard lamination stage to enhance the luminance of their TFT-LCD displays. In addition, many customers have complained about the insufficient luminance of the TFT-LCD displays of the case company. While product engineers have kept tuning the control factors in the soft-to-hard lamination stage through the trial and error method, the improvement of the luminance was not good enough. This study aims to assist the product engineers to fine-tune the settings of the control factors using a new method composed of the Taguchi method, a neural network, and a genetic algorithm. The confirmation experiments showed that the proposed method had increased the average luminance of the TFT-LCD displays from 17.03 to 25.15, which was higher than the required luminance value of 25. As a result, the number of complaints on the TFT-LCD displays had been significantly reduced.

## 1. Introduction

Luminance is an important quality characteristic of a TFT-LCD display. Consumers prefer to have a TFT-LCD display with enough luminance. Several control factors in manufacturing a TFT-LCD display may affect the luminance of a TFT-LCD display. Tuning the control factors using a trial and error method incurs significant overhead in time and resources such as the material and the workforce. Therefore, a more efficient method is required for tuning the control factors in manufacturing TFT-LCD displays. To fine-tune the control factors, we need to perform experiments to find the luminance of TFT-LCD displays for different settings of the control factors. An experiment to find the luminance for a specific parameter setting of the control factors requires about 1.5 months for the case company. To reduce the time in fine-tuning the control factors, we should not perform too many experiments in a new method. In this paper, we first used the traditional Taguchi method to find a control factors’ setting to enhance the luminance of TFT-LCD displays. However, because the Taguchi method allows only a few fixed levels for each control factor, it usually cannot find the global optimal setting for the control factors. Therefore, this paper proposes to improve the Taguchi method by incorporating the Taguchi method with the neural network and the genetic algorithm. In the proposed method, we used the data collected from the Taguchi method to train a neural network to predict the luminance of a TFT-LCD display for a given control factors’ setting. Then, we used a genetic algorithm to search for the global optimal control factors’ setting using the predicted luminance of a control factors’ setting as the fitness value of the setting.

**Figure 1:**
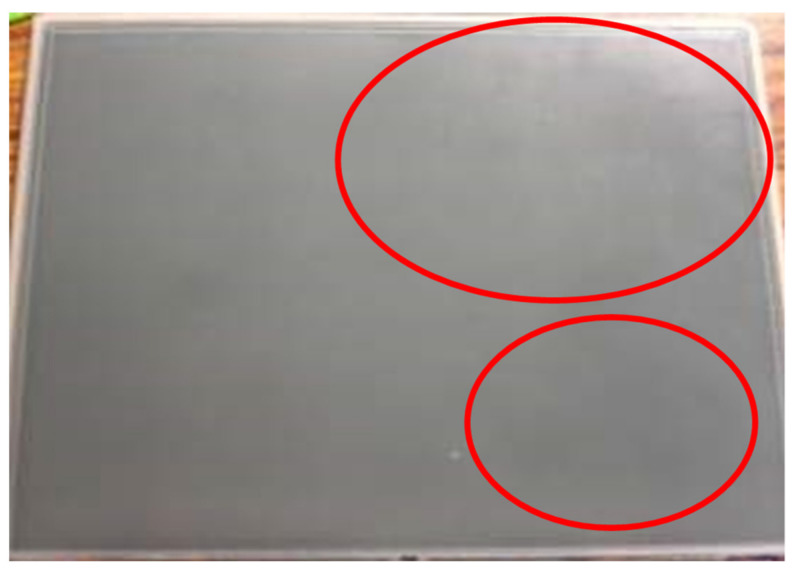
A TFT-LCD display with color streaks.

**Figure 2:**
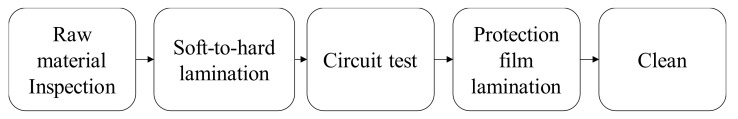
Optical film lamination in the LCM process.

The case company is a TFT-LCD display manufacturer in Taiwan. In performing the optical film lamination step in the liquid crystal module assembly process (LCM), the case company observed color streaks, as shown in Figure 1, on some of their TFT-LCD displays. The color streaks reduced the luminance of the TFT-LCD displays. Therefore, to enhance the luminance of TFT-LCD displays, the optical film lamination step needs to be optimized. The optical film lamination step consists of five stages: the raw material inspection, the soft-to-hard lamination, the circuit test, the protection film lamination, and the clean process, as shown in Figure 2. According to a failure analysis performed by the case company, the color streaks on a TFT-LCD display were mainly due to improper setting of control factors in the soft-to-hard lamination stage. Therefore, this paper focuses on optimizing the soft-to-hard lamination stage to improve the luminance of TFT-LCD displays.

**Figure 3:**
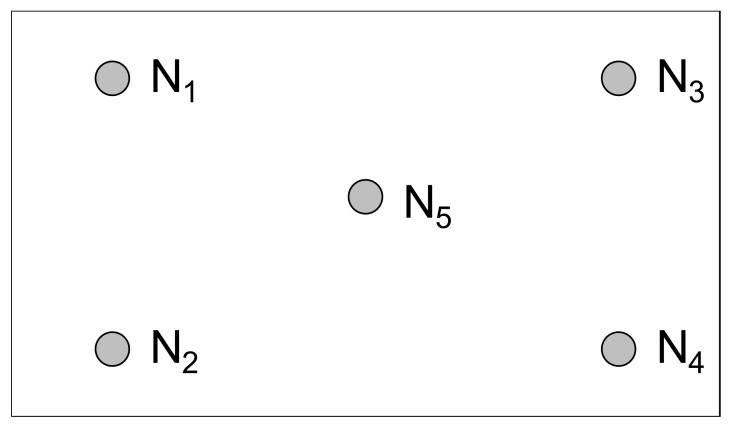
Positions on the substrate to measure the luminance.

**Figure 4:**
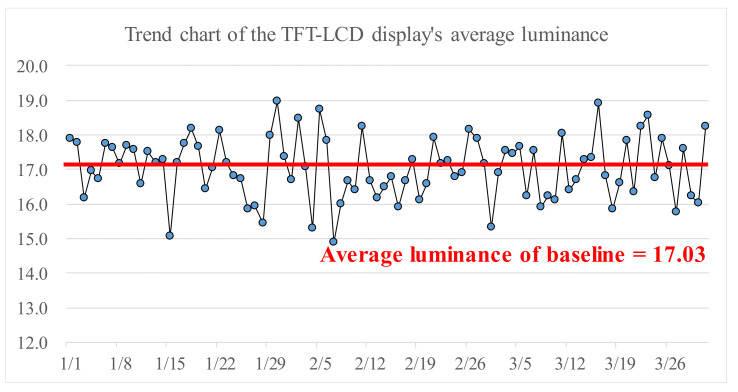
Trend chart of the TFT-LCD display’s average luminance.

For the quality improvement purpose, engineers of the case company selected five positions, as shown in Figure 3, on a TFT-LCD substrate to measure their corresponding values of luminance. The luminance of a TFT-LCD display is the average of the five values of luminance of the selected positions. The case company manufactured about 5000 TFT-LCD displays per day. Therefore, the TFT-LCD displays’ average luminance at a specific date was calculated by averaging the luminance of all the TFT-LCD displays made at that particular date. Figure 4 shows the trend chart for the TFT-LCD displays’ average luminance. The average of the average luminance at all dates in a selected time interval is defined as the baseline, the red line shown in Figure 4. Before fine-tuning the control factors in the sot-to-hard lamination stage, the baseline was 17.03, which was less than the required minimum luminance of 25. Thus, to enhance the TFT-LCD display’s luminance, one needs to find a setting of the control factors such that the baseline is larger than the required minimum luminance of 25. Therefore, we propose in this paper a new method that combines the Taguchi method, a neural network, and a genetic algorithm to fine-tune the setting of control factors in the soft-to-hard lamination stage to enhance the TFT-LCD displays’ luminance.

Many researchers have studied the performance of laminated materials. For example, Lee and Kim [1] investigated the influence of mechanical characteristics on the performance of optical laminating materials in automotive applications by varying the H/vinyl ratio and the hydrogen source ratio. Serhat and Basdogan [2] proposed a multi-objective design methodology to set the parameters’ values in a lamination process to optimize the stiffness of the composite plate with dynamic and load-carrying requirements. Ridhuan et al. [3] studied the effect of interconnecting thickness and yield strength to find maximum peak stress in the longitudinal cross-section of a photovoltaic (PV) laminate during the soldering and lamination processes. For determining the accumulated final residual stresses in a PV laminate, Song et al. [4] simulated the stress evolution of solar cells in manufacturing a conventional silicon wafer-based photovoltaic laminate.

## 2. Literature Review

Since the proposed method combines the Taguchi method, neural networks, and the genetic algorithm to fine-tune the control factors in the lamination stage, we briefly explain the related works and methods in this section.

### 2.1. Manufacturing Processes of TFT-LCD Displays

**Figure 5:**
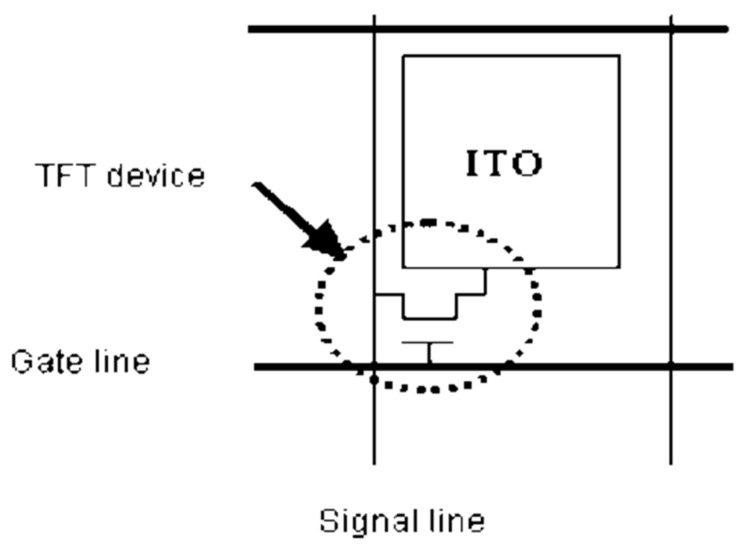
The structure of a TFT-LCD unit.

Thin-film transistor liquid crystal displays (TFT-LCDs) are widely used in many consumer electronic products. The structure of a basic TFT-LCD unit, as shown in Figure 5, consists of a TFT device and a pattern of Indium Tin Oxides (ITO) film-layer for controlling the angle of liquid crystal [5,6]. The TFT device is a switch that controls the number of electrons on the ITO. The TFT device turns on its switch to allow electrons to flow into the ITO. When the number of electrons in the ITO reaches a specific value, the TFT device turns off its switch to trap the electrons in the ITO. A TFT array consisting of millions of TFT-LCD units is the major component of a TFT-LCD display. The other key components of a TFT-LCD display include the glass substrate, the color filter, the polarizer, the driver IC, the liquid crystal (LC), the Polyimide (PI), the Backlight module, the ITO film-layer, and chemical materials. A TFT-LCD uses an active driving mode to control the irradiation of light on the backlight module. The lower polarizer then polarizes the light from the backlight module. Finally, the voltage of an electrode is applied to the liquid crystal to control the angle of the liquid crystal. Different angles of the liquid crystal give different intensities of the polarized light, which pass through the RGB pixels of the color filter to show different luminance and colors on the TFT-LCD display.

The TFT-LCD display’s three major manufacturing processes are the TFT array process, the LCD cell process, and the LC module assembly process. The TFT array process consists of five steps: the gate metal (gate line), the a-Si island (a semiconductor layer), the source (the signal line), the passivation (the insulation layer), and the ITO film-layer (the conductive glass). The LCD cell process assembles the pre-processed TFT array substrate with the color filter substrate and injects liquid crystal between the two substrates. The LCD cell process consists of the PI rubbing step, the one drop filling (ODF) step, the cutting step, and the polarizer lamination step. Finally, the LC module assembly process (LCM) assembles all the required components into a TFT-LCD display. The LCM process consists of the optical film lamination step, driver IC bonding step, the PCB bonding step, the assembly step, and the packing step [5,6].

### 2.2. Taguchi Method

Genichi Taguchi developed a theory for quality control to optimize a system by experimental design. Taguchi’s work, known as the Taguchi method, was based on engineering applications rather than statistics. The Taguchi method has been widely used in the industry for experimental design. For example, Tole et al. [7] used the Taguchi method to find a better combination of engineering parameters to optimize the degree of amorphization (DOA). Lin et al. [8] applied the Taguchi method to simplify the analysis of a product development process. Akyalcin et al. [9] adopted an *L*_9_ orthogonal array to investigate the optimal desilication conditions on mesopore volume.

The Taguchi method can be used for experimental design to improve the quality of a product. At the process design stage, the experimental design aims to determine the setting of control factors to enhance the quality of products. For experimental design efficiency, Taguchi proposed to use an orthogonal array to obtain a complete and reliable experimental result [10]. Table 1 shows an orthogonal array of *L*_12_(2^11^). Columns from *A* to *K* of Table 1 represent different control factors. A value in the orthogonal array is called a level of its corresponding control factor. Table 1 contains eleven columns and twelve rows, indicating that up to eleven control factors and twelve treatments are allocated in the experimental design.

The Taguchi method uses a loss function to measure the quality of a product [10]. When the target value of a product’s quality characteristic is consistent with the actual value of the quality characteristic, the quality loss function is minimized. In other words, the smaller the value of the quality loss function, the better the quality of the product. Taguchi introduced the signal-to-noise ratio (*S/N* ratio) to measure the quality of a setting of the control factors in an experimental design based on the quality loss function. Measured in decibel (dB), an *S/N* ratio considers both the average and the variance of the experimental result. An *S/N* ratio can be defined in three different ways [11]: nominal-the-better, smaller-the-better, and larger-the-better.

The nominal-the-better (NTB) has a specific target value. The quality of a setting of the control factors with NTB is measured by the differences between all the experimental results and the target value. The closer the experimental results to the target value, the better the quality of the experiment is. The *S/N* ratio for the normal-the-better, denoted by $S/N_{NTB}$, is defined in Equation (1), where *m* denotes the target value; *S^2^* denotes the variance of the experimental results; *y_i_* refers to the result of the *ith* experiment; *n* refers to the number of measurements with the same control factors’ setting.

(1) $$S/N_{NTB}=-10\log\left[\frac{\sum_{i=1}^{n}{\left(y_{i}-m\right)}^{2}}{n}\right]=-10\log\left[{\left(\bar{y}-m\right)}^{2}+S^{2}\right]$$

The smaller-the-better (STB) aims for a small target value. The definition of $\mathrm{the}\;S/N_{STB}$ is shown in Equation (2).

(2) $$S/N_{STB}=-10\log\frac{\sum_{i=1}^{n}y_{i}^{2}}{n}=-10\log({\bar{y}}^{2}+S^{2})$$

The larger-the-better (LTB) aims for a large target value. The definition of the $S/N_{LTB}$ is shown in Equation (3).

(3) $$S/N_{LTB}=-10\log\frac{\sum_{i=1}^{n}\frac{1}{y_{i}^{2}}}{n}$$

### 2.3. Neural Network

**Figure 6:**
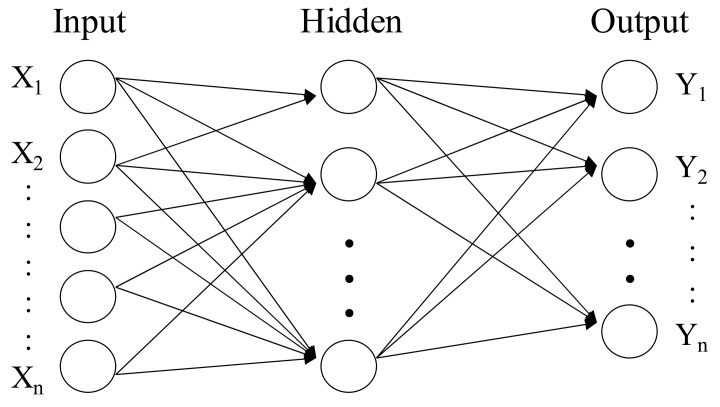
The structure of a three-layered neural network.

A neural network (NN) can be used to construct a model to capture the relationship between the input control factors and the output responses of a manufacturing problem [12,13]. In this paper, we use the multi-layer perceptron, one of the well-known NNs, to construct a model to predict the luminance of a TFT-LCD display given an input of values of different control factors. The neural network is used as the fitness function of a genetic algorithm to find the best setting of the control factors. Figure 6 shows a three-layered NN network where each node in the input layer accepts the value of an input variable, and the output nodes represent the results of the response variables. The input layer is connected to the output layer through one hidden layer. Each link between two nodes in different layers of the NN is associated with an adjustable weight.

To construct an NN, we first specify the structure of the NN and then train the NN with a training dataset. The training of an NN comprises two stages: forward propagation and backward propagation. In the forward propagation stage, inputs are fed into the network to compute the output values. In the backward propagation stage, the differences between the output values and their corresponding actual values are calculated. A loss function based on the differences is defined. Based on the gradient of the loss function, a gradient descent algorithm updates the weights on all the links of the neural network to minimize the loss function [14]. Neural networks have been successfully used in learning the relationship between the input values of the control factors and the output values of the response variables in many manufacturing problems. For example, Mehrpouya et al. [15] used an artificial neural network model to find the optimal laser parameters to join the PET (polyethylene terephthalate) films. Sheikholeslami et al. [16] constructed a neural network to estimate the heat transfer rate in channels for transport fluids in the oil and gas industry. Azizi et al. [17] used an artificial neural network to predict the compressibility factor (z-factor) of natural gases. Ansari et al. [18] trained an artificial neural network to predict the ultimate recovery factor of oil reservoirs by steam-assisted gravity drainage (SAGD).

### 2.4. Genetic Algorithm

A genetic algorithm (GA) imitates the law of survival of the fittest in natural selection. A GA adopts an efficient probabilistic search in a high-dimensional solution space [19]. Many researchers have applied GAs to find solutions to engineering optimization problems. For example, Hosseinabadi et al. [20] adopted a genetic algorithm to solve the open-shop scheduling problem (OSSP). Quan et al. [21] used a genetic algorithm to acquire the relationship between the power, wavelength, current, and temperature from a distributed Bragg reflector laser. Finally, Alipour-Sarabi et al. [22] used a genetic algorithm to minimize the total harmonic distortion of the output signals.

This paper adopted a binary encoding to encode a feasible setting of the control factors into a chromosome. For example, assume that the range of a control factor *X* is [*L_b_*, *U_b_*], and there are *m* digits of significance in the fraction part of *X*. Then, the number of bits in the chromosome of the control factor *X* is determined by Equation (4).

(4) $$\left(U_{b}-L_{b}\right)\times 10^{m}\leq 2^{b}-1\;\to \;b\geq [log_{2}(\left(U_{b}-L_{b}\right)\times 10^{m}+1)]$$

Example: Assuming that $X\in \left[0.15,\;0.75\right],$ the number of significant digits in the fraction part of *X* is 2.

Then, the number of bits for *X,* denoted by *b*, is calculated in the following:

$$b\geq [log_{2}\left(\left(0.75-0.15\right)\times 10^{2}+1\right)]=6$$

In the beginning, the GA randomly generates a set of feasible solutions, each of which is encoded into a chromosome. The collection of all the randomly generated solutions constitutes the first generation of the GA. Then, depending on the application, a fitness function is defined to evaluate the quality of each feasible solution. Finally, the GA iterates itself with three genetic operations: reproduction, crossover, and mutation, to evolve the current generation to the next generation until a chromosome with a satisfying fitness value has been found or a pre-defined maximum number of iterations has been reached. When the GA stops, it returns the best solution of the last population [23].

This paper uses the roulette wheel method to reproduce the child chromosomes from a set of parent chromosomes. The probability for a parental chromosome to be chosen to produce the child chromosomes is proportional to its fitness value.

**Figure 7:**
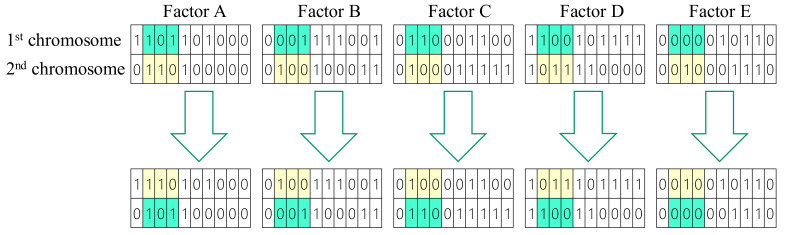
The two-point crossover operation.

We applied a two-point crossover operation on each control factor in the parental chromosomes to generate child chromosomes from a pair of parental chromosomes. Figure 7 shows an illustrative example of five two-point crossover operations on five control factors in a chromosome. In Figure 7, the bit segment from the second position to the fourth position of each control factor at the first chromosome is exchanged with its counterpart in the second chromosome.

## 3. The Proposed Method

This paper first used an orthogonal array to assist the case company in conducting experiments to collect experimental data. Then, it used the Taguchi method to select key control factors for the soft-to-hard lamination stage. Finally, this paper used the Taguchi method to find a better setting for the control factors. However, the pre-defined levels of control factors limited the search space of the Taguchi method. Therefore, the control factors’ setting found by the Taguchi method may not be globally optimal.

**Figure 8:**
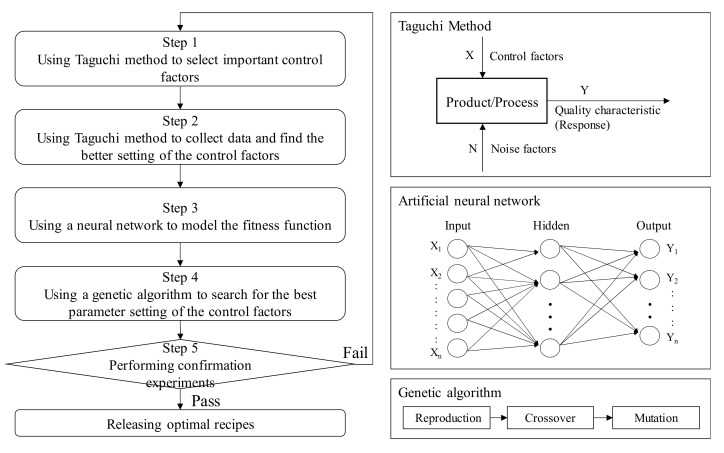
The flowchart of the proposed approach.

Consequently, we use the proposed approach to search for the global optimal setting of the control factors. In the proposed method, we trained an artificial neural network (NN) to predict the luminance of a TFT-LCD display given the input value of each control factor. Then, we used a genetic algorithm (GA) to search for the global optimal setting of the control factors. Finally, we compared the average luminance of the pure Taguchi method with that of our proposed method. Figure 8 shows the flowchart of the proposed method. 

## 4. Case Study

The proposed method consists of five steps:

### 4.1. Using the Taguchi Method to Select Important Control Factors

**Figure 9:**
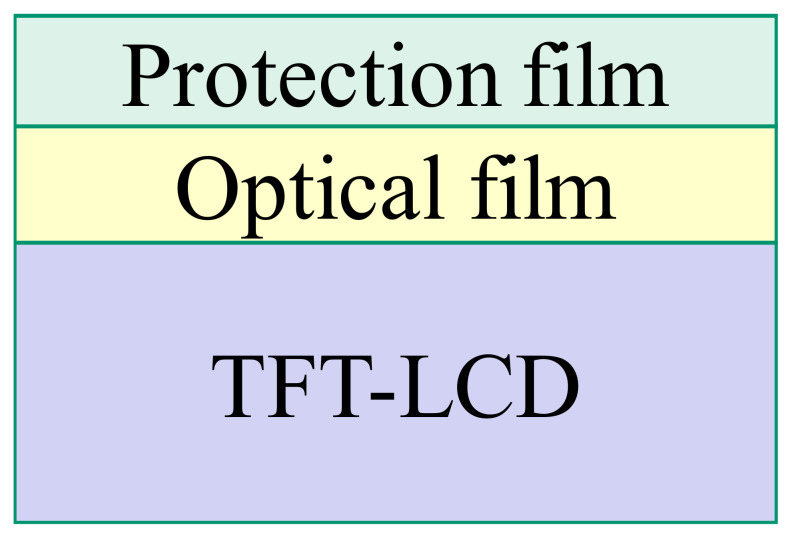
Structure of a TFT-LCD product.

**Figure 10:**
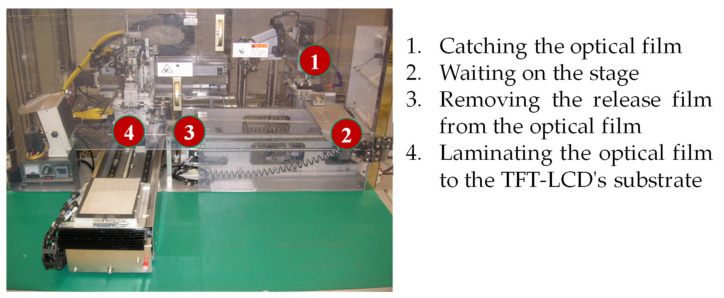
Operations in the soft-to-hard lamination stage.

A TFT-LCD product consists of three layers: the protection film, the optical film, and the TFT-LCD substrate, as shown in Figure 9. As suggested by the engineers of the case company, the improper setting of control factors in the soft-to-hard lamination stage undermines the luminance of a TFT-LCD display. The soft-to-hard lamination stage consists of four operations: catching the optical film, waiting on the stage, removing the release film from the optical film, and laminating the optical film to a TFT-LCD’s substrate as shown in Figure 10. Based on suggestions from on-site engineers, we selected the following eleven control factors for quality improvement: Pre-heating, Roller temperature, Roller wait time, Roller angle, Roller pressure, Roller speed, Dummy, Vacuum pressure, BTW gap, Transfer speed, and Hold time. Table 2 shows the levels and their corresponding values for each control factor.

**Figure 11:**
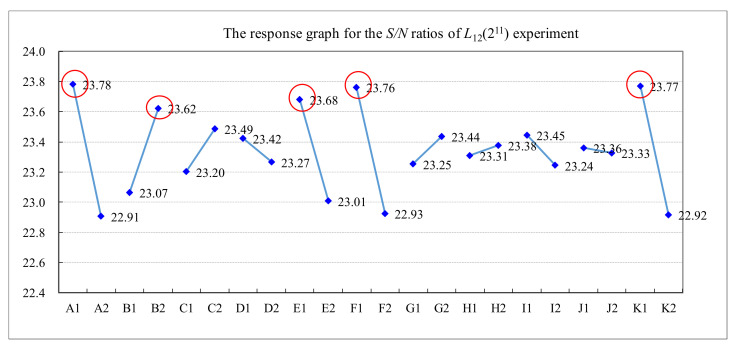
The response graph for the *S/N* ratios of the *L*_12_(2^11^) experiment.

**Figure 12:**
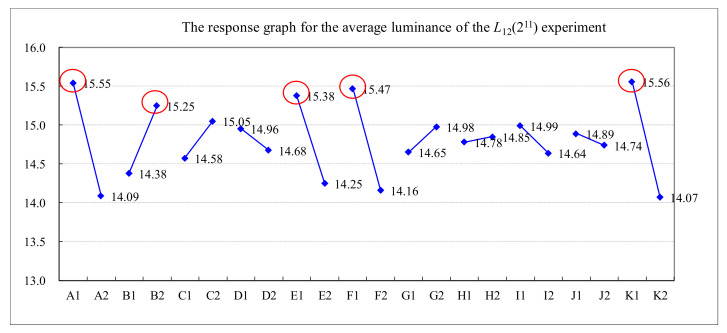
The response graph for the average luminance of the *L*_12_(2^11^) experiment.

This paper used an *L*_12_ (2^11^) orthogonal array in the experimental design for the quality improvement of the TFT-LCD displays. Table 3 shows the five values of measured luminance, from N_1_ to N_5_, of a TFT-LCD display, the average and standard deviation of the five luminance values, and the *S/N* ratio for the experiments with different control factors’ settings. The results of the *S/N* ratio analysis, including the factor response table, the factor response graph, and the analysis of variance (ANOVA), are shown in Table 4, Figure 11, and Table 5, respectively. Table 4 and Figure 11 show the effect of each control factor on the *S/N* ratio. It shows that the order of importance of the control factors is that A (0.87) > K (0.85) > F (0.84) > E (0.67) > B (0.56) > C (0.29) > I (0.20) > G (0.18) > D (0.16) > H (0.07) > J (0.03). Similarly, the results of the luminance analysis are shown in Table 6, Figure 12, and Table 7. Table 6 and Figure 12 show that the order of importance of each control factor on the average luminance is that K (1.48) > A (1.46) > F (1.31) > E (1.13) > B (0.87) > C (0.48) > I (0.35) > G (0.32) > D (0.28) > J (0.15) > H (0.07). From Table 5 and Table 7, the *p*-values of control factors A, B, E, F, and K are less than 0.05. Therefore, control factors A, B, E, F, and K are the key control factors for quality improvement in the soft-to-hard lamination stage.

### 4.2. Using the Taguchi Method to Collect Data and Find the Better Setting of Control Factors

**Figure 13:**
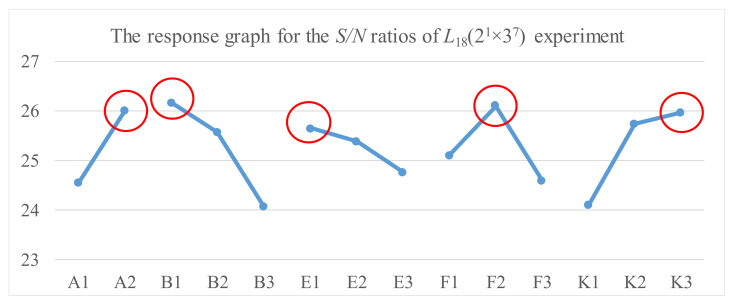
The response graph for the *S/N* ratios of the *L*_18_(2^1^ × 3^7^) experiment.

**Figure 14:**
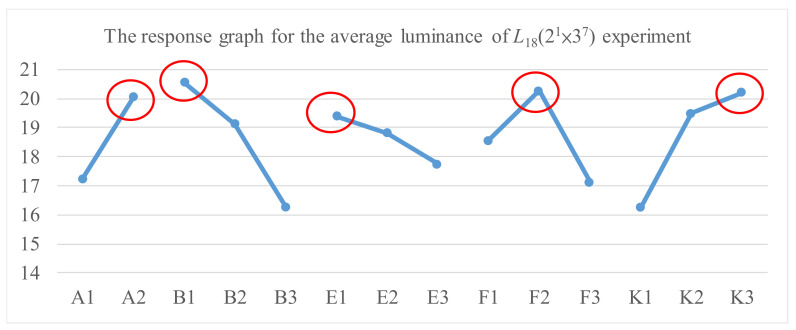
The response graph for the average luminance of the *L*_18_(2^1^ × 3^7^) experiment.

As shown in Figure 8, we used the Taguchi method in step 2 to find a better control factor setting. Table 8 lists the levels and their corresponding values in the experiments for each control factor. We used an *L*_18_(2^1^ × 3^7^) orthogonal array for the experimental design. Table 9 shows the five measured luminance values on a TFT-LCD display, the average and standard deviation of the five luminance values, and the *S/N* ratio for each experiment with different control factors’ settings. The results of the *S/N* ratio analysis, including the factor response table, the factor response graph, and the analysis of variance (ANOVA), are shown in Table 10, Figure 13, and Table 11, respectively. Table 10 and Figure 13 show that the order of importance of control factors on *S/N* ratio is that B (2.11) > K (1.89) > F (1.50) > A (1.45) > E (0.89). Similarly, Table 12, Figure 14, and Table 13 show the results of the luminance analysis. Table 12 and Figure 14 show that the order of importance of the control factors on the average luminance is that B (4.27) > K (3.95) > F (3.14) > A (2.82) > E (1.66). Based on Table 11 and Table 13, the *p*-values of all control factors are less than 0.05. Therefore, all five control factors are important for quality improvement in the soft-to-hard lamination stage. Since the luminance of a TFT-LCD display is the larger, the better, based on Figure 13 and Figure 14, the better setting for control factors is *A_2_, B_1_, E_1_, F_2,_* and *K_3_*, which means that A is set to level 2, B to level 1, E to level 1, F to level 2, and K to level 3 [10]. 

According to [10], the predicted *S/N* ratio $\hat{\eta }$ is calculated using Equation (5), where $\overset{¯}{T}$ is the average of the eighteen *S/N* ratios in Table 9, and ${\overset{¯}{A}}_{2},\;{\overset{¯}{B}}_{1},{\overset{¯}{E}}_{1},{\overset{¯}{F}}_{2}\;and\;{\overset{¯}{K}}_{3}$ are the average *S/N* ratios of *A_2_*, *B_1_*, *E_1_*, *F_2,_* and *K_3_*, respectively.

(5) $$\begin{array}{ll} \hat{\eta } & =\overset{¯}{T}+\left({\overset{¯}{A}}_{2}-\overset{¯}{T}\right)+\left({\overset{¯}{B}}_{1}-\overset{¯}{T}\right)+\left({\bar{E}}_{1}-\overset{¯}{T}\right)+\left({\bar{F}}_{2}-\overset{¯}{T}\right)+\left({\bar{K}}_{3}-\overset{¯}{T}\right)\\ \;={\overset{¯}{A}}_{2}+{\overset{¯}{B}}_{1}+{\bar{E}}_{1}+{\bar{F}}_{2}+{\bar{K}}_{3}-4\overset{¯}{T}\\ \;=25.99+26.18+25.66+26.10+25.97-4\times 25.27=28.815 \end{array}$$

According to Equation (6), the predicted luminance $\overset{⌒}{L}$ is equal to 25.88. Note that in Equation (6), $\overset{¯}{L}$ denotes the average of all the eighteen luminance values in Table 9; $L_{A_{2}}$ denotes the average of the nine luminance values in Table 9 with control factor A equal to level 2. Similarly,$\;L_{B_{1}},L_{E_{1}},L_{F_{2}}and\;L_{K_{3}}$ denote the averages for the corresponding luminance values with *B* = Level 1, *E* = Level 1, *F* = Level 2, and *K* = Level 3, respectively.

(6) $$\begin{array}{ll} \overset{⌒}{L} & =\overset{¯}{L}+\left(L_{A_{2}}-\overset{¯}{L}\right)+\left(L_{B_{1}}-\overset{¯}{L}\right)+\left(L_{E_{1}}-\overset{¯}{L}\right)+\left(L_{F_{2}}-\overset{¯}{L}\right)+\left(L_{K_{3}}-\overset{¯}{L}\right)\\ & =L_{A_{2}}+L_{B_{1}}+L_{E_{1}}+L_{F_{2}}+L_{K_{3}}-4\overset{¯}{L}\\ & =20.06+20.55+19.40+20.27+20.20-4\times 18.65=25.88 \end{array}$$

We conducted a confirmation experiment with three replicated experiments to verify whether the predicted S/N ratio and luminance are acceptable or not. The 95% confidence intervals for both *S/N* ratio and luminance are calculated according to Equation (7) [24] in the following:

(7) $$CI=\sqrt{F_{\alpha ;1,\nu_{2}}\times V_{e}\times \left(\frac{1}{n_{eff}}+\frac{1}{r}\right)}$$

where

$F_{\alpha ;1,\nu_{2}}$ = the F ratio;

α = risk. The confidence level = 1 − α;

ν_2_ = degrees of freedom for pooled error variance;

*V_e_* = pooled error variance;

*n_eff_* = Effective number of observations

(8) $$n_{eff}=\frac{Total\;number\;of\;experiments}{1+\left[total\;degrees\;of\;freedom\;associated\;with\;items\;used\;in\;estimating\;mean\right]};$$

*r* = number of replicated experiments.

The 95% confidence intervals of the *S/N* ratio and luminance are calculated as follows:

$$n_{eff}=\frac{18}{1+\nu_{A}+\nu_{B}+\nu_{E}+\nu_{F}+\nu_{K}}=\frac{18}{1+1+2+2+2+2}=1.8;\;CI_{SN}=\sqrt{F_{0.05;1.8}\times V_{e}\times \left(\frac{1}{n_{eff}}+\frac{1}{r}\right)}=\sqrt{5.32\times 0.19\times \left(\frac{1}{1.8}+\frac{1}{3}\right)}=0.948;\;CI_{Luminance}=\sqrt{F_{0.05;1.8}\times V_{e}\times \left(\frac{1}{n_{eff}}+\frac{1}{r}\right)}=\sqrt{5.32\times 0.9149\times \left(\frac{1}{1.8}+\frac{1}{3}\right)}=2.08;$$

Accordingly, the 95% confidence interval for the *S/N* ratio is 28.815 ± 0.948, and the 95% confidence interval for the luminance is 25.88 ± 2.08. Table 14 shows that the average *S/N* ratio is 27.94 and the average luminance is 24.98 for the confirmation experiment. Both figures fall into their corresponding confidence intervals, indicating that the predictions made by the Taguchi method are accurate. 

Table 15 shows that the control factors’ setting found by the Taguchi method has offered an improvement of 46.67 percentage in luminance (from 17.03 to 24.98) and an improvement of 13.53 percentage in the *S/N* ratio (from 24.61 to 27.94).

Since the Taguchi method allows only a few fixed levels for each control factor, it may not find the global optimal setting for all the control factors. We, therefore, use a genetic algorithm to search for the global optimal setting for the control factors.

### 4.3. Using a Neural Network to Model the Fitness Function

**Figure 15:**
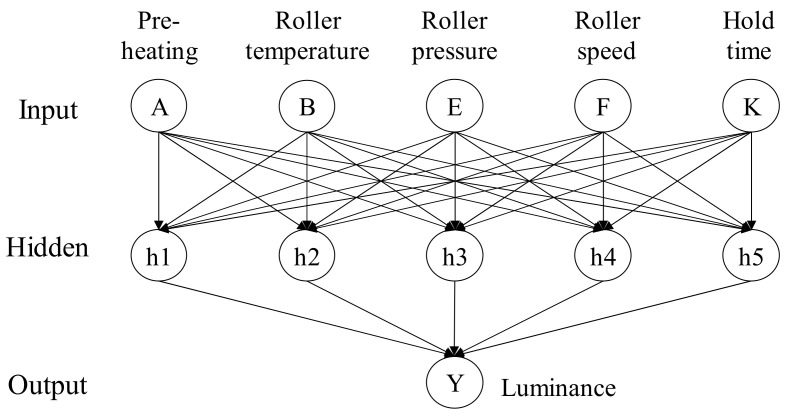
The NN structure for the soft-to-hard lamination stage.

To construct an effective genetic algorithm, we need to define a suitable fitness function. In this paper, we choose to use a neural network model to predict the fitness value for a specific setting of the control factors. We used the dataset in Table 9 to train a neural network model to compute the luminance of a TFT-LCD display, given a specific setting of the control factors. The average luminance and the control factors’ values together constitute a training example in the dataset. We then randomly selected eighty percent examples from the dataset as the training dataset and the rest as the testing dataset. The proposed neural network in Figure 15 has five input nodes, one hidden layer, and one output node. We used ReLU as the activation function and set the maximum number of iteration to 1000 as the termination condition.

Furthermore, we set the learning rate and the momentum to 0.1 and 0.9, respectively. Finally, we constructed nine different neural networks with varying numbers of hidden nodes to determine the best neural network architecture. We then chose the neural network with five hidden nodes for the fitness function since it produced the smallest root-mean-squared-error on predicting the testing dataset, as shown in Table 16. 

### 4.4. Using a Genetic Algorithm to Search for the Global Optimal Setting for the Control Factors

In the proposed GA, we first encoded the value of each input factor into a bit string, as discussed in Section 2.4. We then set the population size to 100, used the roulette wheel method for reproduction, and applied a two-point crossover for the crossover operation. Finally, we set the crossover rate to 0.9, the mutation rate to 0.05, and the maximum iteration to 1000. 

**Figure 16:**
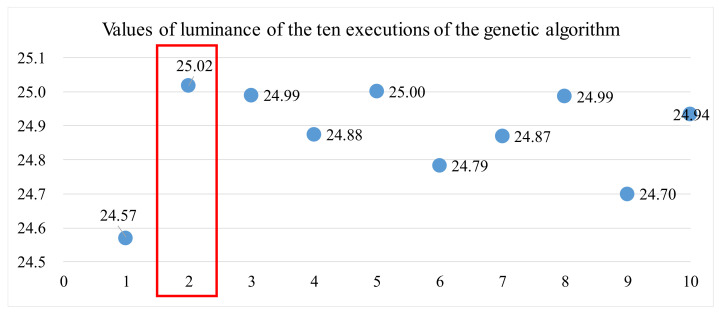
Luminance values of the ten executions of the genetic algorithm.

Table 17 summarizes the luminance values of the ten executions of the GA. Figure 16 depicts the luminance for each execution and shows that the maximum luminance is 25.02. Therefore, the global optimal control factors’ setting predicted by the GA is that A = 29 °C, B = 50 °C, E = 0.15 kg/cm^2^, F = 2500 mm/min, and K = 57 msec.

### 4.5. Performing Confirmation Experiments

We conducted three confirmation experiments to verify the feasibility of the global optimal setting found by the GA Table 18 shows the luminance of the confirmation experiments with A = 29 °C, B = 50 °C, E = 0.15 kg/cm^2^, F = 2500 mm/min, and K = 57 msec. Table 19 shows the average luminance of the original data, the Taguchi method, and the proposed method, respectively. The confirmation experiments showed an average luminance of 25.15, which is very close to 25.02, the maximum luminance predicted by the GA. Table 19 shows that the GA has offered a 47.68 percentage improvement in luminance and a 13.82 percentage improvement in the S/N ratio compared to the original data.

## 5. Conclusions

The case company suffered from color streaks on its TFT-LCD displays in their LCM manufacturing process. This paper proposed a new method to set the control factors’ values to enhance the TFT-LCD displays’ luminance. We first used the Taguchi method to collect on-site manufacturing data for quality improvement [10,11]. Then, we used the analysis of variance (ANOVA) to find the key control factors in the soft-to-hard lamination stage for improving the luminance of a TFT-LCD display [10]. The selected key control factors are Pre-heating (A), Roller temperature (B), Roller pressure (E), Roller speed (F), and Hold time (K). Afterward, we used the Taguchi method to determine the setting of the key control factors for enhancing the TFT-LCD displays’ luminance. The Taguchi method has found a setting of the key control factors that promoted the luminance of a TFT-LCD display from 17.03 to 24.98, which is slightly less than the required luminance of 25. However, because the Taguchi method allows only a few fixed levels for each control factor, it usually cannot find the global optimal setting for the control factors [10]. Therefore, we proposed to use a neural network to predict the luminance for a given control factors’ setting [12,13]. With the predicted luminance for each control factors’ setting, we used a GA to search for the global optimal setting of the control factors [23]. The proposed GA has found a control factors’ setting, which is better than the Taguchi method. The confirmation experiments showed that the proposed GA method had increased the luminance of a TFT-LCD display from 17.03 to 25.15, which was higher than the required luminance of 25. After improving the luminance of the TFT-LCD displays, the case company has increased its annual revenue by USD 950,000.

## Figures and Tables

**Table 1:** *L*_12_(2^11^) orthogonal array.

Treatment	A	B	C	D	E	F	G	H	I	J	K
1	1	1	1	1	1	1	1	1	1	1	1
2	1	1	1	1	1	2	2	2	2	2	2
3	1	1	2	2	2	1	1	1	2	2	2
4	1	2	1	2	2	1	2	2	1	1	2
5	1	2	2	1	2	2	1	2	1	2	1
6	1	2	2	2	1	2	2	1	2	1	1
7	2	1	2	2	1	1	2	2	1	2	1
8	2	1	2	1	2	2	2	1	1	1	2
9	2	1	1	2	2	2	1	2	2	1	1
10	2	2	2	1	1	1	1	2	2	1	2
11	2	2	1	2	1	2	1	1	1	2	2
12	2	2	1	1	2	1	2	1	2	2	1

**Table 2:** Control factors and their corresponding levels for the *L*_12_(2^11^) experiment.

Factor	Pre-Heating (°C)	Roller Temperature (°C)	Roller Wait Time (Sec)	Roller Angle (Degree)	Roller Pressure (kg/cm^2^)	Roller Speed (mm/min)	Dummy (pcs)	Vacuum Pressure	BTW Gap (mm)	Transfer Speed (mm/sec)	Hold Time (msec)
	A	B	C	D	E	F	G	H	I	J	K
Lv 1	50	40	3	80	0.7	5000	2	2.8	5	4000	60
Lv 2	70	50	5	85	0.8	6000	4	3	10	5000	70

**Table 3:** *L*_12_(2^11^) orthogonal array and data for the Taguchi method experiment.

EXP.	Control Factors											Luminance					Average Luminance	Standard Deviation	*S/N*
	A	B	C	D	E	F	G	H	I	J	K	N_1_	N_2_	N_3_	N_4_	N_5_			
1	1	1	1	1	1	1	1	1	1	1	1	17.61	16.77	17.45	16.99	16.30	17.03	0.53	24.61
2	1	1	1	1	1	2	2	2	2	2	2	13.34	15.12	12.70	14.91	14.57	14.13	1.06	22.94
3	1	1	2	2	2	1	1	1	2	2	2	14.18	14.30	14.66	13.92	13.48	14.11	0.44	22.98
4	1	2	1	2	2	1	2	2	1	1	2	15.45	15.70	15.61	15.72	14.52	15.40	0.50	23.74
5	1	2	2	1	2	2	1	2	1	2	1	16.69	15.60	15.15	16.53	15.28	15.85	0.71	23.98
6	1	2	2	2	1	2	2	1	2	1	1	17.93	16.46	17.03	17.22	15.16	16.76	1.04	24.44
7	2	1	2	2	1	1	2	2	1	2	1	16.28	16.56	15.90	15.80	15.52	16.01	0.41	24.08
8	2	1	2	1	2	2	2	1	1	1	2	13.92	12.49	12.19	12.23	11.38	12.44	0.92	21.84
9	2	1	1	2	2	2	1	2	2	1	1	13.41	11.85	12.04	11.91	13.63	12.57	0.88	21.94
10	2	2	2	1	1	1	1	2	2	1	2	15.68	15.24	14.97	15.63	14.24	15.15	0.59	23.59
11	2	2	1	2	1	2	1	1	1	2	2	12.86	12.99	13.24	13.87	13.11	13.22	0.39	22.41
12	2	2	1	1	2	1	2	1	2	2	1	14.50	15.26	14.41	16.28	15.20	15.13	0.75	23.57

**Table 4:** Factor response table for the *S/N* ratios of the *L*_12_(2^11^) experiment.

Factor	A	B	C	D	E	F	G	H	I	J	K
Level 1	23.78	23.07	23.20	23.42	23.68	23.76	23.25	23.31	23.45	23.36	23.77
Level 2	22.91	23.62	23.49	23.27	23.01	22.93	23.44	23.38	23.24	23.33	22.92
Effect	0.87	0.56	0.29	0.16	0.67	0.84	0.18	0.07	0.20	0.03	0.85
Rank	1	5	6	9	4	3	8	10	7	11	2

**Table 5:** ANOVA on the *S/N* ratios of the *L*_12_(2^11^) experiment.

Source	DF	SS	MS	F-Value	*p*-Value
A	1	2.297	2.297	24.61	0.003
B	1	0.934	0.934	10	0.019
C	1	0.244 *	–	–	–
D	1	0.076 *	–	–	–
E	1	1.357	1.357	14.53	0.009
F	1	2.099	2.099	22.48	0.003
G	1	0.102 *	–	–	–
H	1	0.014 *	–	–	–
I	1	0.121 *	–	–	–
J	1	0.003 *	–	–	–
K	1	2.183	2.183	23.39	0.003
Error	(6)	(0.5601)	(0.09335)	–	–
Total	11	534.71	–	–	–
				R-Sq	R-Sq(adj)
				94.1%	89.1%

* pooling into the error term.

**Table 6:** Factor response table for the average luminance of the *L*_12_(2^11^) experiment.

Factor	A	B	C	D	E	F	G	H	I	J	K
Level 1	15.55	14.38	14.58	14.96	15.38	15.47	14.65	14.78	14.99	14.89	15.56
Level 2	14.09	15.25	15.05	14.68	14.25	14.16	14.98	14.85	14.64	14.74	14.07
Effect	1.46	0.87	0.48	0.28	1.13	1.31	0.32	0.07	0.35	0.15	1.48
Rank	2	5	6	9	4	3	8	11	7	10	1

**Table 7:** ANOVA on values of the average luminance of the *L*_12_(2^11^) experiment.

Source	DF	SS	MS	F-Value	*p*-Value
A	1	6.3739	6.37388	22.72	0.003
B	1	2.2755	2.27552	8.11	0.029
C	1	0.6828 *	–	–	–
D	1	0.2325 *	–	–	–
E	1	3.8466	3.84656	13.71	0.01
F	1	5.1493	5.14933	18.35	0.005
G	1	0.3167 *	–	–	–
H	1	0.015 *	–	–	–
I	1	0.3683 *	–	–	–
J	1	0.0684 *	–	–	–
K	1	6.6032	6.60323	23.53	0.003
Error	(6)	(1.684)	(0.2806)	–	–
Total	11	25.932	–	–	–
				R-Sq	R-Sq(adj)
				93.51%	88.10%

* pooled into the error terms.

**Table 8:** Control factors and their corresponding levels for the *L*_18_(2^1^ × 3^7^) experiment.

Factor	Pre-Heating (°C)	Roller Temperature (°C)	Roller Pressure (kg/cm^2^)	Roller Speed (mm/min)	Hold Time (msec)
	A	B	E	F	K
Level 1	25	50	0.15	2500	20
Level 2	50	65	0.45	4000	40
Level 3	–	80	0.75	5500	60

**Table 9:** *L*_18_(2^1^ × 3^7^) orthogonal array and data for the Taguchi method experiment.

EXP.	Control Factors					Luminance					Average Luminance	Standard Deviation	*S/N*
	A	B	E	F	K	N_1_	N_2_	N_3_	N_4_	N_5_			
1	1	1	1	1	1	17.32	17.23	17.04	17.23	17.62	17.29	0.21	24.75
2	1	2	2	2	2	19.74	19.90	19.74	19.79	19.66	19.77	0.09	25.92
3	1	3	3	3	3	13.98	13.74	13.87	13.64	13.58	13.76	0.16	22.77
4	1	2	2	3	3	18.59	18.44	18.58	18.55	18.43	18.52	0.08	25.35
5	1	3	3	1	1	11.78	11.64	11.48	11.59	11.71	11.64	0.11	21.32
6	1	1	1	2	2	22.84	22.58	22.80	23.31	22.81	22.87	0.27	27.18
7	1	1	3	2	3	21.49	21.34	21.62	21.08	21.05	21.32	0.25	26.57
8	1	2	1	3	1	14.34	15.18	14.46	14.85	14.70	14.71	0.33	23.34
9	1	3	2	1	2	14.97	15.56	15.06	15.49	15.35	15.29	0.26	23.68
10	2	3	2	2	1	16.94	16.98	17.04	16.86	16.92	16.95	0.07	24.58
11	2	1	3	3	2	20.27	19.80	19.42	19.29	18.66	19.49	0.60	25.79
12	2	2	1	1	3	21.34	23.01	20.89	21.13	21.26	21.52	0.85	26.64
13	2	3	1	3	2	18.89	18.84	18.31	19.12	18.63	18.76	0.31	25.46
14	2	1	2	1	3	25.04	24.61	24.88	24.46	25.19	24.84	0.30	27.90
15	2	2	3	2	1	19.38	19.64	19.47	19.30	19.38	19.43	0.13	25.77
16	2	2	3	1	2	20.94	20.81	20.65	20.99	20.56	20.79	0.18	26.35
17	2	3	1	2	3	21.49	21.27	21.23	20.98	21.34	21.26	0.19	26.55
18	2	1	2	3	1	17.64	17.42	17.99	17.38	17.07	17.50	0.34	24.98

**Table 10:** Factor response table for the *S/N* ratios of the *L*_18_(2^1^ × 3^7^) experiment.

Level	A	B	E	F	K
1	24.54	26.18	25.66	25.11	24.10
2	25.99	25.56	25.38	26.10	25.73
3	–	24.06	24.76	24.60	25.97
Effect	1.45	2.11	0.89	1.50	1.86
Rank	4	1	5	3	2

**Table 11:** ANOVA on the *S/N* ratios of the *L*_18_(2^1^ × 3^7^) experiment.

Source	DF	SS	MS	F	*p*-Value
A	1	9.404	9.4045	49.49	0
B	2	14.2	7.1001	37.36	0
E	2	2.514	1.2571	6.61	0.02
F	2	6.983	3.4914	18.37	0.001
K	2	12.332	6.1659	32.44	0
Error	8	1.52	0.19	–	–
Total	17	46.954	–	–	–
				R-Sq	R-Sq(adj)
				96.76%	93.12%

**Table 12:** Factor response table for the average luminance of the *L*_18_(2^1^ × 3^7^) experiment.

Level	A	B	E	F	K
1	17.24	20.55	19.40	18.56	16.25
2	20.06	19.12	18.81	20.27	19.49
3	–	16.28	17.74	17.12	20.20
Effect	2.82	4.27	1.66	3.14	3.95
Rank	4	1	5	3	2

**Table 13:** ANOVA on values of the average luminance of the *L*_18_(2^1^ × 3^7^) experiment.

Source	DF	SS	MS	F	*p*-Value
A	1	35.815	35.815	39.15	0
B	2	56.797	28.3983	31.04	0
E	2	8.522	4.2609	4.66	0.046
F	2	29.708	14.8542	16.24	0.002
K	2	53.229	26.6146	29.09	0
Error	8	7.319	0.9149	–	–
Total	17	191.39	–	–	–
				R-Sq	R-Sq(adj)
				96.18%	91.87%

**Table 14:** Results of the confirmation experiments for the Taguchi Method.

EXP.	Control Factors					Luminance					Average Luminance	Standard Deviation	*S/N*
	A	B	E	F	K	N_1_	N_2_	N_3_	N_4_	N_5_			
1	2	1	1	2	3	26.58	23.89	23.53	25.09	24.75	24.77	1.192	27.85
2	2	1	1	2	3	24.48	26.09	24.50	25.36	25.17	25.12	0.668	27.99
3	2	1	1	2	3	24.98	25.08	24.62	25.62	25.04	25.05	0.370	28.01
Total average											24.98	0.743	27.94

**Table 15:** The average luminance of TFT-LCD displays before and after the Taguchi method.

Comparison	Pre-Heating (°C)	Roller Temperature (°C)	Roller Pressure (kg/cm^2^)	Roller Speed (mm/min)	Hold Time (msec)	Average Luminance	*S/N*
	(A)	(B)	(E)	(F)	(K)		
Before improvement	50	40	0.7	5000	60	17.03	24.61
Taguchi method	50	50	0.15	4000	60	24.98	27.94
Improvement						46.67%	13.53%

**Table 16:** Candidate neural networks.

NN Structure	5-2-1	5-3-1	5-4-1	5-5-1	5-6-1	5-7-1	5-8-1	5-9-1	5-10-1
Training RMSE	0.037	0.005	0.005	0.005	0.005	0.005	0.005	0.005	0.005
Testing RMSE	0.138	0.119	0.105	0.090	0.102	0.127	0.113	0.102	0.094

Note: Learning rate = 0.1; momentum = 0.9; number of epochs = 1000.

**Table 17:** Summary on values of luminance of the ten executions of the GA.

Item	The Largest Luminance	The Smallest Luminance	Average	Standard Deviation
Luminance	25.02	24.57	24.87	0.148

**Table 18:** Confirmation experiments for the genetic algorithm.

EXP.	Luminance					Average Luminance	Standard Deviation	*S/N*
	N_1_	N_2_	N_3_	N_4_	N_5_			
1	25.08	25.11	25.31	25.18	25.23	25.181	0.092	28.02
2	25.08	25.08	25.11	25.08	25.03	25.075	0.028	27.98
3	25.50	25.24	24.90	25.25	25.07	25.195	0.224	28.03
Total average						25.150	0.115	28.01

**Table 19:** The luminance of the original data, the Taguchi method, and the GA.

Comparison	Pre-Heating (°C)	Roller Temperature (°C)	Roller Pressure (kg/cm^2^)	Roller Speed (mm/min)	Hold Time (msec)	Average Luminance	*S/N*
	(A)	(B)	(E)	(F)	(K)		
Before improvement	50	40	0.7	5000	60	17.03	24.61
Taguchi methods	50	50	0.15	4000	60	24.98	27.94
Proposed method	29	50	0.15	2500	57	25.15	28.01
Improvement						47.68%	13.82%

## Data Availability

The data presented in this study are available on request from the corresponding author.
